# Supplementary material for: Triplet–triplet upconversion enhanced by spin–orbit coupling in organic light-emitting diodes
Source: Nat Commun. 2019 Nov 21;10:5283. doi: 10.1038/s41467-019-13044-1 (PMC6872538; doi:10.1038/s41467-019-13044-1)
Supplement: Supplementary file 1 — Supplementary Information [file 41467_2019_13044_MOESM1_ESM.doc]

**Triplet–triplet upconversion enhanced by spin–orbit coupling in organic light-emitting diodes**

Ryota Ieuji^1,2^, Kenichi Goushi^1,2,3^*, and Chihaya Adachi^1,2,3^*

^1^Department of Applied Chemistry and Center for Organic Photonics and Electronics Research (OPERA), Kyushu University, 744 Motooka, Nishi, Fukuoka 819-0395, Japan

^2^Japan Science and Technology Agency (JST), Exploratory Research for Advanced Technology (ERATO), Adachi Molecular Exciton Engineering Project, Kyushu University, 744 Motooka, Nishi-ku, Fukuoka 819-0395, Japan

^3^International Institute for Carbon Neutral Energy Research (WPI-I^2^CNER), Kyushu University, 744 Motooka, Nishi-ku, Fukuoka 819-0395, Japan

*Corresponding authors’ e-mail:

E-mail: goushi@opera.kyushu-u.ac.jp and adachi@opera.kyushu-u.ac.jp

**Supplementary Figures**

**Supplementary Figure. S1 | Molecular orbitals calculated by TD-DFT calculations.**  Molecular orbitals related to the ^1^CT (S_1_), ^1^LE, and ^3^LE (T_1_) states of the anthracene derivatives (see also **Supplementary Table S1**).

**Fig. S2** (a) Absorption and fluorescence spectra of the anthracene derivatives in different solvents. (b) Stokes shifts of the anthracene derivatives as a function of the orientation polarizability calculated by **Equation S3**. The solid lines are the fitting results by the Lippert-Mataga equation.

**Fig. S3** Phosphorescence spectra of the anthracene derivatives (2 wt%) dispersed in a polymer matrix. Spectra were integrated between 10 and 16 ms after 360nm excitation at room temperature. Spectra are offset for clarity.

**Fig. S4** Energy levels and twice the T_1_ energies (dashed line) of the anthracene derivatives calculated using B3LYP/6-31G(d), where n is the quantum number used to indicate the excited state.

**Supplementary Figure. S5** **2 | EL spectra.** EL spectra of OLEDs with the anthracene derivatives as emitters, along with PL spectra for comparison. **a** DPA, **b** DMAC-σ-ANT, **c** DMAC-σ-ANTCN, **d** PXZ-σ-ANT, **e** PXZ-σ-ANTCN, and **f** ANT-TRZ. The device structure is ITO (100 nm) / α-NPD (20 nm) / TAPC (20 nm) / anthracene derivative (20 nm) / TPBi (40 nm) / LiF (1 nm) / Al (80 nm).

**Supplementary Fig. ure S6** **3 | *J*-*V* characteristics.** Current density (*J*)–voltage (*V*) characteristics of the OLEDs containing anthracene derivatives. The device structure is ITO (100 nm) / α-NPD (20 nm) / TAPC (20 nm) / anthracene derivative (20 nm) / TPBi (40 nm) / LiF (1 nm) / Al (80 nm).

**Supplementary Figure. S7** **4 | *η*_EQE_-*J* characteristics.** External quantum efficiency (*η*_EQE_)–current density (*J*) characteristics of the OLEDs containing anthracene derivatives. The device structure is ITO (100 nm) / α-NPD (20 nm) / TAPC (20 nm) / anthracene derivative (20 nm) / TPBi (40 nm) / LiF (1 nm) / Al (80 nm).

**Supplementary Figure. S5 | Pulse width dependences.8** Dependence of the radiative-exciton production efficiency (*η*_r_) estimated from transient EL characteristics at *J*=1 mA/cm^2^ on pulse width. **a** DPA, **b** DMAC-σ-ANT, **c** DMAC-σ-ANTCN, **d** PXZ-σ-ANT, **e** PXZ-σ-ANTCN, and **f** ANT-TRZ. The device structure is ITO (100 nm) / α-NPD (20 nm) / TAPC (20 nm) / anthracene derivative (20 nm) / TPBi (40 nm) / LiF (1 nm) / Al (80 nm).

**Supplementary Figure 6 | Possible TTU transitions of DPA.**

Molecular orbitals related to TTU transitions from ^3^(TT) to S_n_ in DPA.

**Supplementary Figure 7 | Possible TTU transitions of DMAC-σ-ANT.** Molecular orbitals related to TTU transitions from ^3^(TT) to S_n_ in DMAC-σ-ANT.

**Supplementary Figure 8 |** **Possible TTU transitions of DMAC-σ-ANTCN.** Molecular orbitals related to TTU transitions from ^3^(TT) to S_n_ in DMAC-σ-ANTCN.

**Supplementary Figure 9 |** **Possible TTU transitions of PXZ-σ-ANT.** Molecular orbitals related to TTU transitions from ^3^(TT) to S_n_ in PXZ-σ-ANT.

**Supplementary Figure 10 |** **Possible TTU transitions of PXZ-σ-ANTCN.**

Molecular orbitals related to TTU transitions from ^3^(TT) to S_n_ in PXZ-σ-ANTCN.

**Supplementary Figure 11 |** **Possible TTU transitions of ANT-TRZ.**

Molecular orbitals related to TTU transitions from ^3^(TT) to S_n_ in ANT-TRZ.

**Supplementary Tables**

**Supplementary Table 1.** Electronic transitions of the anthracene derivatives calculated using B3LYP/6-31G(d).

.

**Supplementary Table 2.** Absorption and fluorescence peak wavelengths of the anthracene derivatives in different solvents.

^a)^*f* is the orientation polarizability calculated by Equation S3. ^b)^*λ*_A_ is the absorption peak at the long-wavelength side. ^c)^*λ*_f_ is the emission peak at the short-wavelength side. When ^1^CT with a tiny contribution ^1^LE was observed, the emission peak of ^1^CT was employed in the Lippert-Mataga plot.

^a)^The maximum electroluminescence wavelength with the excitation wavelength of 360nm. ^b)^The photoluminescence quantum yield of the thin film under argon atmosphere with the excitation wavelength of 360 nm. ^c)^The device structures are: (A) ITO (100 nm) / α-NPD (20 nm) / TAPC (20 nm) / EML (20 nm) / TPBi (40 nm) / LiF (1 nm) / Al (80 nm), (B) ITO (100 nm) / HAT-CN (10 nm) / Tris-PCz (30 nm) / EML (20 nm) / TmPyPB (40 nm) / LiF (1 nm) / Al (80 nm), and (C) ITO (100 nm) / HAT-CN (10 nm) / Tris-PCz (30 nm) / EML (20 nm) / TPBi (40 nm) / LiF (1 nm) / Al (80 nm), respectively. ^d)^The maximum electroluminescence wavelength. ^e)^Commission internationale de l'Eclairage (CIE) recorded at 200 mA/cm^2^. ^f)^The maximum external electroluminescence efficiency. ^g)^The maximum current efficiency. ^h)^The maximum power efficiency.

**Supplementary Table 3.** Electronic transitions calculated by B3LYP/6-31G(d) of S*_n_* with energies lower than twice the T_1_ energy (~3.5 eV) and T*_m_* with energies in the range of 3.45–3.50 eV.


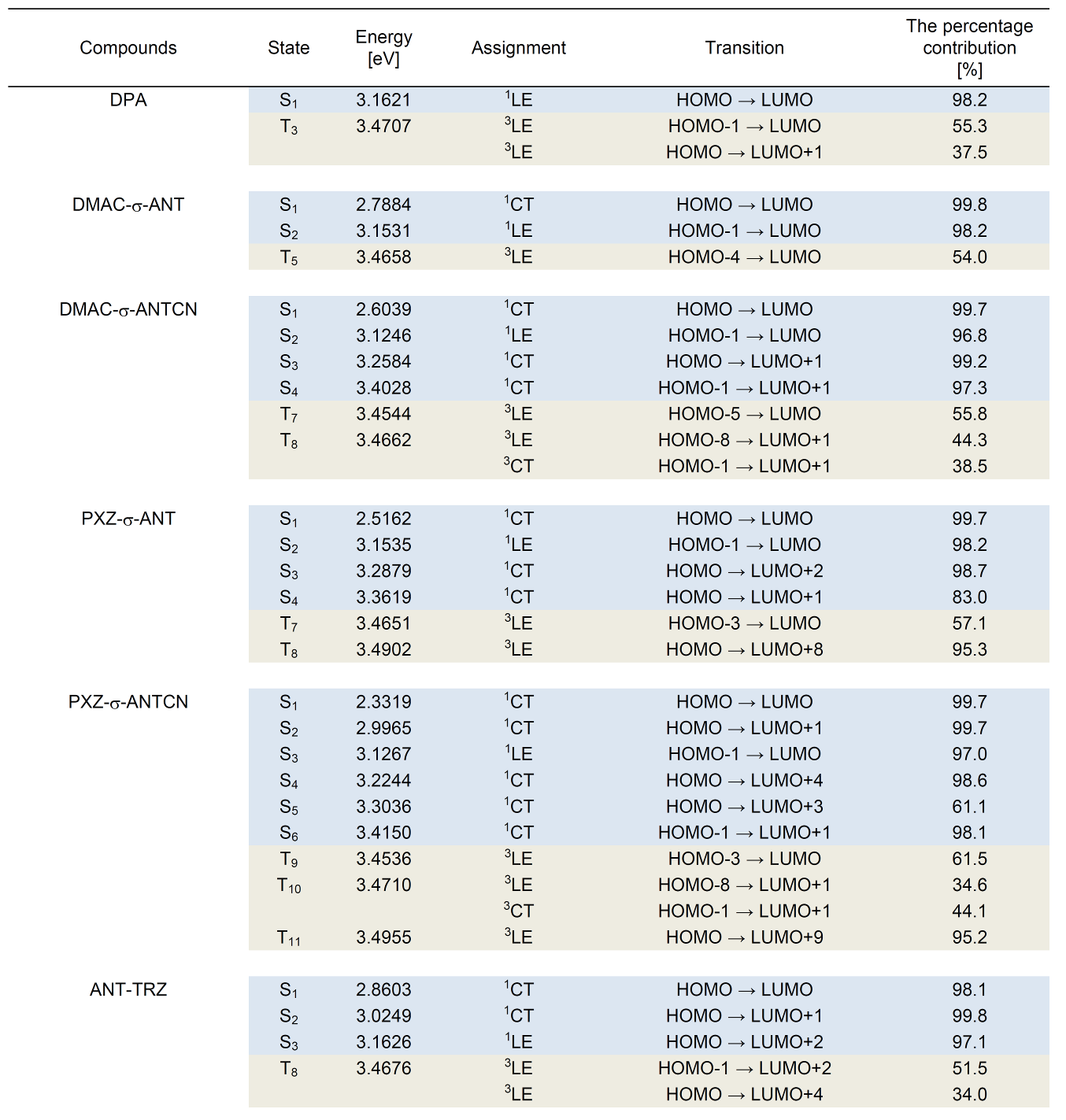


**Supplementary Note 1: Synthesis and characterization**

**Synthesis and characterization data for** **9,10-dihydro-9,9-dimethyl-10-(4-(10-phenylanthracen-9-yl)phenyl)acridine (DMAC-σ-ANT)**

9-(4-Bromophenyl)-10-phenylanthracene (3.00 g, 7.30 mmol), 9,10-dihydro-9,9-dimethylacridine (2.00 g, 9.55 mmol), potassium carbonate (3.00 g, 21.9 mmol), palladium(II) acetate (0.16 g, 0.73 mmol), and tri-*tert*-butylphosphonium tetrafluoroborate (0.21 g, 0.73 mmol) were mixed in a flask containing nitrogen-saturated xylene (30 mL). The mixture was stirred and heated under reflux for 15 h. The cooled mixture was partitioned between dichloromethane and water. The organic layer was separated. The combined organic layers were dried with MgSO_4_ and then evaporated. The residue was purified by column chromatography using hexane and dichloromethane as eluents. The yield of DMAC-σ-ANT was 82% (3.23 g).

[NMR]

^1^H NMR (500 MHz, CDCl_3_): δ = 7.84 (d, *J* = 8.5 Hz, 2H), 7.75 (d, *J* = 8.0 Hz, 4H), 7.63–7.57 (m, 5H), 7.53–7.51 (m, 4H), 7.46 (t, *J* = 7.0 Hz, 2H), 7.39 (t, *J* = 8.0 Hz, 2H), 7.13 (t, *J* = 8.5 Hz, 2H), 7.00 (t, *J* = 7.5 Hz, 2H), 6.59 (d, *J* = 4.8 Hz, 2H), 1.75 (s, 6H) ppm. ^13^C NMR (125 MHz, CDCl_3_) δ = 141.4, 140.9, 139.4, 139.3, 137.9, 136.4, 134.2, 131.8, 131.7, 130.6, 130.3, 130.2, 128.8, 127.9, 127.5, 127.0, 126.8, 125.7, 125.6, 125.5, 121.1, 114.5 ppm.

[Elemental analysis]

Calcd for C_41_H_31_N: C, 91.58; H, 5.81; N, 2.60. Found: C, 91.67; H, 5.79; N, 2.67.

**Synthesis and characterization data for** **4-(10-(4-(9,9-dimethylacridin-10(9*H*)-yl)phenyl)anthracen-9-yl)benzonitrile (DMAC-σ-ANTCN)**

4-[(10-(4-Chlorophenyl)-9-anthracenyl]benzonitrile (1.00 g, 2.57 mmol), 9,10-dihydro-9,9-dimethylacridine (0.805 g, 3.85 mmol), potassium carbonate (1.06 g, 7.67 mmol), palladium(II) acetate (0.06 g, 0.28 mmol), and tri-*tert*-butylphosphonium tetrafluoroborate (0.07 g, 0.24 mmol) were mixed in a flask containing nitrogen-saturated toluene (25 mL). The mixture was stirred and heated under reflux for 15 h. The cooled mixture was partitioned between dichloromethane and water. The organic layer was separated. The combined organic layers were dried with MgSO_4_ and then evaporated. The residue was purified by column chromatography using hexane and dichloromethane as eluents. The yield of DMAC-σ-ANTCN was 24% (0.35 g).

[NMR]

^1^H NMR (500 MHz, CDCl_3_): δ = 7.95 (d, *J* = 8.5 Hz, 2H), 7.86 (d, *J* = 8.5 Hz, 2H), 7.73 (d, *J* = 8.5 Hz, 2H), 7.66 (d, *J* = 8.0 Hz, 2H), 7.61–7.58 (m, 4H), 7.53 (d, *J* = 9.0 Hz, 2H), 7.48 (t, *J* = 8.3 Hz, 2H), 7.43 (t, *J* = 8.3 Hz, 2H), 7.13 (t, *J* = 8.5 Hz, 2H), 7.01 (t, *J* = 8.3 Hz, 2H), 1.75 (s, 6H) ppm. ^13^C NMR (125 MHz, CDCl_3_) δ = 141.4, 139.0, 137.6, 134.1, 132.7, 132.6, 131.9, 130.7, 130.1, 129.8, 127.3, 126.8, 126.6, 126.2, 126.0, 125.6, 121.1, 114.4, 112.1 ppm.

[Elemental analysis]

Calcd for C_42_H_30_N_2_: C, 89.65; H, 5.37; N, 4.98. Found: C, 89.47; H, 5.38; N, 5.00.

**Synthesis and characterization data for** **10-(4-(10-phenylanthracen-9-yl)phenyl)-10*H*-phenoxazine (PXZ-σ-ANT)**

9-(4-Bromophenyl)-10-phenylanthracene (3.00 g, 7.30 mmol), phenoxazine (2.02 g, 11.0 mmol), potassium carbonate (3.00 g, 21.9 mmol), palladium(II) acetate (0.16 g, 0.73 mmol), and tri-*tert*-butylphosphonium tetrafluoroborate (0.21 g, 0.73 mmol) were mixed in a flask containing nitrogen-saturated xylene (30 mL). The mixture was stirred and heated under reflux for 15 h. The cooled mixture was partitioned between dichloromethane and water. The organic layer was separated. The combined organic layers were dried with MgSO_4_ and then evaporated. The residue was purified by column chromatography using hexane and dichloromethane as eluents. The yield of PXZ-σ-ANT was 73% (2.73 g).

[NMR]

^1^H NMR (500 MHz, CDCl_3_): δ = 7.77–7.71 (m, 6H), 7.64–7.56 (m, 5H), 7.51 (d, *J* = 7.5 Hz, 2H), 7.43 (t, *J* = 7.5 Hz, 2H), 7.38 (t, *J* = 7.5 Hz, 2H), 6.77–6.70 (m, 6H), 6.21 (d, *J* = 7.5 Hz, 2H) ppm. ^13^C NMR (125 MHz, CDCl_3_) δ = 144.5, 139.8, 139.3, 138.6, 138.0, 136.2, 134.8, 134.5, 131.6, 131.2, 130.3, 130.1, 128.8, 127.9, 127.5, 126.9, 125.8, 125.5, 123.7, 121.8, 115.9, 113.7 ppm.

[Elemental analysis]

Calcd for C_38_H_25_NO: C, 89.21; H, 4.93; N, 2.74. Found: C, 89.29; H, 4.94; N, 2.79.

**Synthesis and characterization data for** **4-(10-(4-(10*H*-phenoxazin-10-yl)phenyl)anthracen-9-yl)benzonitrile (PXZ-σ-ANTCN)**

4-[(10-(4-Chlorophenyl)-9-anthracenyl]benzonitrile (1.75 g, 4.50 mmol), phenoxazine (1.24 g, 6.75 mmol), potassium carbonate (2.00 g, 14.4 mmol), palladium(II) acetate (0.10 g, 0.45 mmol), and tri-*tert*-butylphosphonium tetrafluoroborate (0.13 g, 0.45 mmol) were mixed in a flask containing nitrogen-saturated toluene (20 mL). The mixture was stirred and heated under reflux for 15 h. The cooled mixture was partitioned between dichloromethane and water. The organic layer was separated. The combined organic layers were dried with MgSO_4_ and then evaporated. The residue was purified by column chromatography using hexane and dichloromethane as eluents. The yield of PXZ-σ-ANTCN was 85% (2.07 g).

[NMR]

^1^H NMR (500 MHz, CDCl_3_): δ = 7.94 (d, *J* = 8.0 Hz, 2H), 7.78 (d, *J* = 9.0 Hz, 2H), 7.70 (d, *J* = 8.0 Hz, 2H), 7.65 (d, *J* = 8.0 Hz, 2H), 7.61 (d, *J* = 8.0 Hz, 2H), 7.58 (d, *J* = 8.5 Hz, 2H), 7.48–7.40 (m, 4H), 6.77–6.71 (m, 6H), 6.20 (d, *J* = 9.0 Hz, 2H) ppm. ^13^C NMR (125 MHz, CDCl_3_) δ = 144.7, 144.5, 139.4, 134.7, 134.3, 132.7, 132.6, 131.4, 130.1, 129.8, 127.2, 126.6, 126.2, 126.0, 123.7, 121.9, 116.0, 113.7 ppm.

[Elemental analysis]

Calcd for C_39_H_24_N_2_O: C, 87.29; H, 4.51; N, 5.22. Found: C, 87.48; H, 4.41; N, 5.33.

**Synthesis and characterization data for** **2,4-diphenyl-6-(4-(10-phenylanthracene-9-yl)phenyl)-1,3,5-triazine (ANT-TRZ)**

4,4,5,5-Tetramethyl-2-[4-(10-phenylanthracen-9-yl)phenyl]-1,3,2-dioxaborolane (3.0 g, 6.57 mmol), 2-chloro-4,6-diphenyl-1,3,5-triazine (2.64 g, 9.86 mmol), potassium carbonate (1.82 g, 13.2 mmol), and tetrakis(triphenylphosphine)palladium(0) (0.38 g, 0.33 mmol) were mixed in a flask containing nitrogen-saturated toluene (60 mL), ethanol (15 mL), and H_2_O (15 mL). The mixture was stirred and heated at 80 °C for 15 h. The cooled mixture was partitioned between chloroform and water. The organic layer was separated. The combined organic layers were dried with MgSO_4_ and then evaporated. The residue was purified by column chromatography using hexane and dichloromethane as eluents. The yield of ANT-TRZ was 86% (3.17 g).

[NMR]

^1^H NMR (500 MHz, CDCl_3_): δ = 9.03 (d, *J* = 8.0 Hz, 2H), 8.86 (d, *J* = 6.5 Hz, 4H), 7.78–7.76 (m, 2H), 7.74–7.72 (m, 4H), 7.65–7.60 (m, 8H), 7.57 (t, *J* = 7.4 Hz, 1H), 7.51 (d, *J* = 7.3 Hz, 2H), 7.38–7.35 (m, 4H) ppm. ^13^C NMR (125 MHz, CDCl_3_) δ = 172.0, 137.7, 136.4, 135.8, 132.8, 132.0, 131.5, 130.0, 129.8, 129.2, 128.9, 128.6, 127.7, 127.2, 126.9, 125.5, 125.3 ppm.

[Elemental analysis]

Calcd for C_41_H_27_N_3_: C, 87.67; H, 4.85; N, 7.48. Found: C, 87.70; H, 4.82; N, 7.45.
